# Impact of child development at primary school entry on adolescent health—protocol for a participatory systematic review

**DOI:** 10.1186/s13643-021-01694-6

**Published:** 2021-05-07

**Authors:** Michelle Black, Amy Barnes, Mark Strong, David Taylor-Robinson

**Affiliations:** 1 School of Health and Related Research, The University of Sheffield, Regent Court, 30 Regent Street, Sheffield, S1 4DA UK; 2 Public Health, Policy and Systems, Institute of Population Health, University of Liverpool, Liverpool, L69 3GL UK

**Keywords:** Child development, Primary School, Adolescent health, Inequality, Public health

## Abstract

**Background:**

Reducing child health inequalities is a global health priority and evidence suggests that optimal development of knowledge, skills and attributes in early childhood could reduce health risks across the life course. Despite a strong policy rhetoric on giving children the ‘best start in life’, socioeconomic inequalities in children’s development when they start school persist. So too do inequalities in child and adolescent health. These in turn influence health inequalities in adulthood. Understanding how developmental processes affect health in the context of socioeconomic factors as children age could inform a holistic policy approach to health and development from childhood through to adolescence. However, the relationship between child development and early adolescent health consequences is poorly understood. Therefore the aim of this review is to summarise evidence on the associations between child development at primary school starting age (3–7 years) and subsequent health in adolescence (8–15 years) and the factors that mediate or moderate this relationship.

**Method:**

A participatory systematic review method will be used. The search strategy will include; searches of electronic databases (MEDLINE, PsycINFO, ASSIA and ERIC) from November 1990 onwards, grey literature, reference searches and discussions with stakeholders. Articles will be screened using inclusion and exclusion criteria at title and abstract level, and at full article level. Observational, intervention and review studies reporting a measure of child development at the age of starting school and health outcomes in early adolescence, from a member country of the Organisation for Economic Co-operation and Development, will be included. The primary outcome will be health and wellbeing outcomes (such as weight, mental health, socio-emotional behaviour, dietary habits). Secondary outcomes will include educational outcomes. Studies will be assessed for quality using appropriate tools. A conceptual model, produced with stakeholders at the outset of the study, will act as a framework for extracting and analysing evidence. The model will be refined through analysis of the included literature. Narrative synthesis will be used to generate findings and produce a diagram of the relationship between child development and adolescent health.

**Discussion:**

The review will elucidate how children’s development at the age of starting school is related to subsequent health outcomes in contexts of socioeconomic inequality. This will inform ways to intervene to improve health and reduce health inequality in adolescents. The findings will generate knowledge of cross-sector relevance for health and education and promote inter-sectoral coherence in addressing health inequalities throughout childhood.

**Protocol Registration:**

This systematic review protocol has been registered with PROSPERO CRD42020210011.

**Supplementary Information:**

The online version contains supplementary material available at 10.1186/s13643-021-01694-6.

## Background

Reducing child health inequalities is a global health priority and evidence suggests that optimal development of knowledge, skills and attributes in early childhood could reduce health risks from childhood through to adulthood [1]. Positive child development in the early years (age 0–3 years) brings about wide ranging human capital development in later life which strongly influences wellbeing, obesity, mental health, heart disease, literacy and numeracy, criminality and economic productivity [2]. This evidence for investment in early years on human capital development and the resultant economic gains in later life [3, 4], together with the evidence for the early years as a critical period of development [5], make it a prime area for public policy and public health investment. However, current policy (‘best start in life’) and research on health and development has neglected children from age 5 years to adolescence, and there is scope for research and action on child health and development in this period to evolve from an emphasis on the first 1000 days and ‘school readiness’ to the first 8000 days in order to support development needs across children’s life cycle [6]. Understanding how developmental processes affect health in the context of socioeconomic factors as children age could inform a holistic policy approach to health and development from childhood through to adolescence.

Recognising the interconnected nature of health and development in childhood, and the importance of socioeconomic circumstance in determining outcomes, many programmes are in place across the UK which seek to address health and development across the wider determinants of child health, such as quality early years education [7], universal services such as welfare and health visiting [8], parenting programmes [9] and community support through children’s centres [10, 11]. Whilst improvements for children as a whole are being seen for some health outcomes (asthma, epilepsy, diabetes) [12], inequalities in child health are not reducing, with inequalities in outcomes in relation to socioeconomic status [12] and indeed inequalities in some outcomes are widening [13]. This is particularly the case for obesity and mental ill health in early adolescence [14] with negative consequences for weight [15] and wellbeing [16] in adulthood. Socioeconomic inequalities in child development are also apparent. Analysis of the Millennium Cohort Study (a nationally representative cohort set to follow the lives of over 18,000 children born in the year 2000) found that UK children from low- to middle-income families were 5 months behind children from high-income families in terms of vocabulary skills and had more behavioural problems at age 5 years [17]. These inequalities in early child development and health tend to tack forward and increase over time to influence inequalities in later health outcomes [18].

There is evidence that programmes which encompass parenting support and early learning opportunities in or out of the home enhance child development in readiness for school improving cognitive and non-cognitive skills in children [19]. Positive cognitive development on starting school is associated with academic achievement by age 13 years [20] and socio-emotional development by age 10 years [21]. Non-cognitive skills such as social skills and self-regulation on starting school also improve academic success and psychosocial outcomes in subsequent years [22]. Whilst the beneficial effects of education on health in adulthood acquired through knowledge, work and social status are clear [23], there is less evidence of the effect of early child development interventions on health outcomes in childhood; other than limited evidence for obesity reduction, greater social competence, improved mental health and crime prevention [24] and on reducing childhood hospitalisations for infections and injury [25]. So there is evidence that programmes to enhance child development in readiness for school improve academic success, socio-emotional and psychosocial outcomes but the evidence for whether and how measures of child development impact subsequent health in childhood is limited.

Child development on starting school is defined in this study as cognitive or physical or linguistic or socio-emotional development at school starting age. There is evidence that measures of cognitive development at primary school starting age, as a component part of a model incorporating routinely collected data, predict socio-emotional behaviour and obesity at age 11 years [26]. Moving beyond the predictive value of measures to understanding early education as a developmental process in a social context [27] is important if we are to understand how emerging social and cognitive pathways in children interconnect with pathways stemming from socioeconomic circumstances. To improve child health and address inequality, evidence is needed on the mediating pathways between child development on starting school and these later child health outcomes and the socioeconomic and environmental factors which shape this relationship [28].

There is evidence that family stress, material living circumstances and parental behaviours are the main pathways stemming from socioeoconomc circumstance which lead to inequalities in child health [29]. These factors are potential modifiers of the relationship between child development on starting school and adolescent health. A modifier is a variable which alters the strength of association between an exposure and an outcome. In addition to understanding what might affect the strength of the relationship, it is important to understand what variables may explain the relationship. Identifying direct pathways between child development and health (such as knowledge/literacy and cognitive/social pathways) aids understanding of mediators of the relationship. A mediator is a variable which explains the association between and exposure and an outcome.

Increasing understanding of the pathways between child development and health is pertinent learning for improving health because it is the interactions between early childhood development and the biological and social changes during mid-childhood, shaped by socioeconomic factors that influence health-related behaviours in adolescents [30]. However, the relationship between child development and early adolescent health consequences is poorly understood. Better understanding this relationship could provide knowledge on targeted public health interventions in primary school age children and provide a focus for action and policy coherence across the health and education sectors; and help to mitigate the effect of detrimental socioeconomic factors on child development on later health outcomes and inequalities in those outcomes. Therefore, the aim of this review is to summarise evidence on the associations between child development at primary school starting age (3–7 years) and subsequent health in adolescence (11–15 years) and the factors that mediate or moderate this relationship.

## Method

### Protocol registration

The present protocol has been registered within the PROSPERO database (registration number CRD42020210011) and is being reported in accordance with the reporting guidance provided in the Preferred Reporting Items for Systematic Reviews and Meta-Analyses Protocols (PRISMA-P) statement [31, 32] (see checklist in Additional file 1). The planned review will be reported according to the Preferred Reporting Items for Reporting Systematic Reviews and Meta-Analyses (PRISMA) 2020 Statement [33, 34]

### Review questions

#### The planned review will address the following questions:

- What are the associations between measures of child development recorded at primary school starting age (3–7 years) and subsequent health in adolescence (8–15 years)?
- What are the effect modifiers (socioeconomic and environmental factors) of this relationship? (This will identify variables which alter the strength of the observed associations.)
- What are the mediators of this relationship? (This will identify variables or pathways which explain the observed associations.)

### Study design

We will undertake a participatory systematic review, involving engagement with national and local stakeholders across health and education sectors. Participation will occur in the following ways: after an initial scoping search and review of papers, discussions with stakeholders will take place to identify any further relevant studies and to develop an initial conceptual model. This initial conceptual model will act as a framework for extracting and analysing evidence identified in the systematic review. The model will be revised and refined through analysis of the included literature. Narrative synthesis will be used to generate findings and produce a diagram of the relationship between child development in the early years of primary school and adolescent health outcomes. This participatory review method adds value over traditional review methods when clarifying underlying theory, ensuring all valued outcomes are captured, adding insight to relationships between outcomes and understanding of how, when and where interventions may work [35]. Participatory methods to produce diagrams, maps or models help to uncover theories of change and assumptions underpinning pathways between cause and effect [36]. They are increasingly recognised for their potential to make a contribution to systematic review methodology [37] and particularly in the field of public health [38].

### Information sources and search strategy

MEDLINE, PsycINFO, ASSIA and ERIC will be searched for results from November 1990 onwards. The reference lists from all included articles will be searched for eligible articles that may have been missed by the electronic search. Further relevant literature will be identified through stakeholder discussions. Grey literature searching will be undertaken by searching relevant organisations websites and discussions with stakeholders, to find all relevant literature for inclusion. The search terms relate to measures of child development in the early years of primary school and health outcomes in early adolescence. Studies will be limited to those that include children, some or all of whom are aged between 3 and 15 years and those that are in English. A pilot search strategy has been undertaken (Additional file 2).

### Data management

Dates of searches and results will be recorded using Excel. Search results will be downloaded to EndNote desktop software. Studies identified through reference searching, stakeholder discussions and grey literature will be recorded and imported into EndNote

### Eligibility criteria

#### Definition of terms

In this review, child development refers to a measure of cognitive or physical or linguistic or socio-emotional development at primary school starting age (3–7 years).

### Inclusion criteria

Observational studies (ecological, case-control, cohort (prospective and retrospective)) RCTs, quasi experimental, review level studies including theory papers which are the following:

- Studies of children that include a measure of child development at age 3–7 (the age most children enter pre-school or school) and weight/mental health outcomes between age 8 and 15 years.
- Studies that explore factors which affect associations between child development and these outcomes
- Studies that explore mechanisms or pathways between child development and these outcomes

Cross-sectional studies, conference abstracts, dissertations and studies reporting neither outcomes data nor mechanism will be excluded.

The population and context, exposure, outcomes and study designs are described below and summarised in relation to inclusion and exclusion criteria in Table 1.

**Table 1:** Summary of eligibility criteria

	Inclusion	Exclusion
**Population and context**	Studies must include children, some or all of whom are aged between 3 and 15 years, across socioeconomic strata in high-income country settings, defined as OECD membership.	Studies of children from non-OECD countries. Studies which focus solely on a particular subset of children with a particular health or development need.
**Exposure**	A measure of child development at primary school starting age (3–7 years), defined as cognitive or physical or linguistic or socio-emotional development at school starting age, measured by any of the following: • School readiness, as measured by scales such as the Bracken Basic Concepts Scale Revised (BBCS-R) [39] • Cognitive development as measured by, for example, non-reading intelligence tests, vocabulary tests, maths tests or parent/teacher ratings. • Language and literacy (as measured by academic achievement test scores such as pre-reading/reading, vocabulary, oral comprehension, phonological awareness, pre-writing/writing or verbal skills. • Emotional well-being and social competence (as measured by behavioural assessments of social interaction, problem behaviours, social skills and competencies, child-parent relationship/child-teacher relationship). • Physical development. Studies that explore socioeconomic and environmental factors which affect associations between child development at primary school starting age and these outcomes Studies that explore mechanisms or pathways between child development at primary school starting age and these outcomes.	Studies reporting neither data nor mechanism between exposure and outcome will be excluded.
**Outcome**	**Primary outcome(s)** The review will incorporate evidence health and wellbeing outcomes, reported between the ages of 8–15 years, specifically: Weight (BMI) Mental Health (as measured by standard questionnaires or clinically) Socio-emotional behaviour Proxy measures such as dietary habits and behaviour and measures of wellbeing will be included. **Secondary outcome(s)** Educational outcomes Performance at the end of primary school (age 10–11), measured by standardized tests.	Studies reporting neither data nor mechanism between exposure and outcome will be excluded.
**Study design and sources**	Observational studies (ecological, case-control, cohort (prospective and retrospective)) RCTs, quasi experimental, review level studies including theory papers	Cross-sectional studies, conference abstracts, books, dissertations, opinion piece

### Population and context

Studies must include children, some or all of whom are aged between 3 and 15 years, across socioeconomic strata in high-income country settings, defined as OECD membership.

### Exposure

A measure of child development at primary school starting age (3–7 years), defined as cognitive or physical or linguistic or socio-emotional development at school starting age, including

- School readiness, as measured by scales such as the Bracken Basic Concepts Scale Revised (BBCS-R) [39] and Good Level of Development
- Cognitive development as measured by, for example, non-reading intelligence tests, vocabulary tests, maths tests or parent/teacher ratings.
- Language and literacy (as measured by academic achievement test scores such as pre-reading/reading, vocabulary, oral comprehension, phonological awareness, pre-writing/writing or verbal skills.
- Emotional well-being and social competence (behavioural assessments of social interaction, problem behaviours, social skills and competencies, child-parent relationship/child-teacher relationship), measured using the Child Behaviour Checklist.
- Physical development as measured by amount of physical activity or assessment of gross motor skills.

## Primary outcome(s)

The primary outcomes of interest will be weight and mental health as quantitative data, including measures of wellbeing. The outcomes measures are the following:

- Weight (BMI)
- Mental health (as measured by standard questionnaires or clinically)
- Socio-emotional behaviour (as measured by social competence, emotional competence behavioural problems, self-regulation and executive function)
- Proxy measures such as dietary habits and behaviour and measures of wellbeing will be included.

These outcome measures were highlighted in an initial scoping review of the literature and during discussions with stakeholders.

### Secondary outcome(s)

The secondary outcome of interest is educational outcomes measured as:

- Performance at the end of primary school (age 10–11), measured by standardized tests.

The rationale for this outcome is that it facilitates analysis through consideration of possible temporal dynamics to the relationship under study.

### Development of a conceptual model

We have undertaken a scoping review to identify the main factors and pathways between child development at primary school starting age (3–7 years) and subsequent health outcomes at age 8–15 years. Meetings with five stakeholders from local authority, health, education and voluntary sector were held in September 2020 to explore perspectives on these pathway areas; considering in particular, the following:

- How health outcomes in adolescence are most affected by socioeconomic circumstances in child development at the start of primary school
- General perceptions of what the mediating pathways are, including how pathways are connected and feedback loops
- Where in the system would intervening have most impact on socioeconomic inequality in child development on later health outcomes in adolescence

**Fig. 1:**
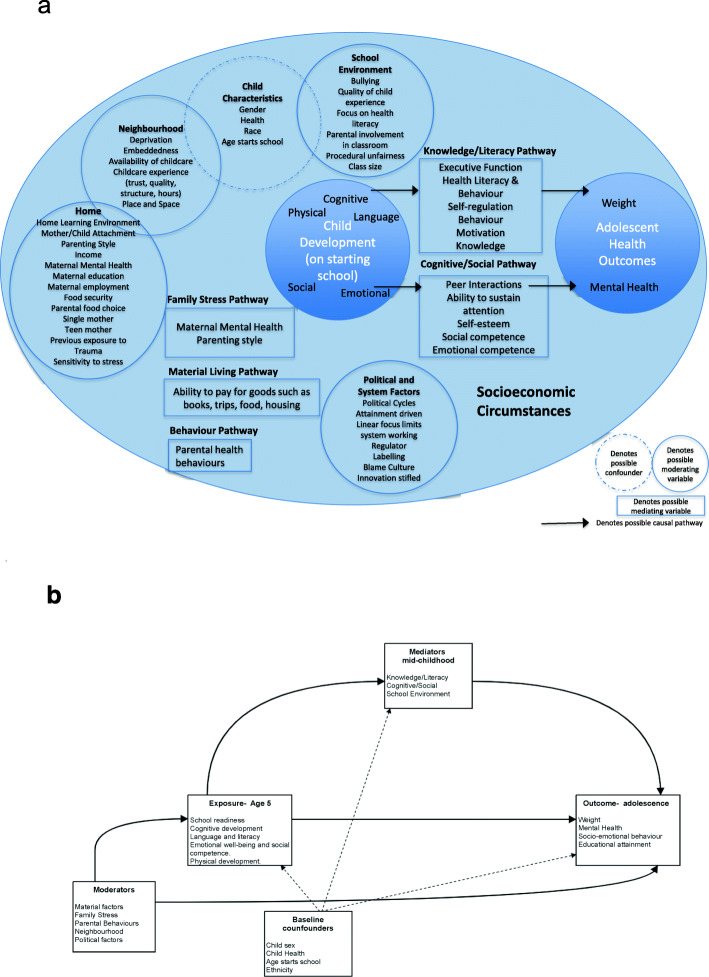
**a** Conceptual model. How does development in the early years of primary school age children affect health in adolescence in the context of socioeconomic inequality? early-childhood to early adolescence (age 3–15).:**b** Illustrative DAG of the relationship between child development in the early years of primary school and adolescent health

Participatory methods and tools, including concept mapping approaches will continue to be used in stakeholder meetings to finalise a conceptual model of the pathways (see Fig. 1a for draft). This initial model forms a framework for the review and provides initial categories for extracting and analysing evidence from published studies. The model will then be revised and refined iteratively through analysis of the included literature to produce a final diagram. This will illustrate where factors in the initial diagram were not reported in the literature and where there may be associations and relationships between factors. The model will be used to formulate a directed acyclic graph (DAG) for further statistical analysis of the associations and pathways in subsequent phase of this study (see Fig. 1b).

### Selection and data collection process

Articles will be screened using the inclusion and exclusion criteria at title and abstract level, and then at full article level by the review team. At each stage, a sample will be checked independently by another member of the review team and inter-rater reliability will be recorded. Any queries regarding inclusion will be discussed with at least one other team member. Data extraction using a bespoke form will be undertaken for all studies that meet the inclusion criteria by the lead reviewer and a sample will be checked independently by another team member. A data extraction form (Additional file 3) has been developed using previous expertise of the team and has been piloted on a sample of different sources. The following data will be extracted: study design, country, year, study population, study characteristics, child development measure, health outcomes, factors affecting associations, pathways, main findings, strengths and weaknesses. In cases where additional data from studies is required, the lead reviewer will contact the study authors.

### Quality assessment

Quality assessment of the included studies will be conducted using the Liverpool University Quality Assessment Tool (LQAT), which allows for a specific tool to be used for each study design [40]. This tool has been independently evaluated against other quality assessment tools [41]. Quality assessments will be done by the main author and second checked by a member of the review team and any discrepancies will be discussed.

### Strategy for data synthesis

This review is broad in scope and as such it is anticipated that there will be considerable heterogeneity between studies in terms of design and measurements of the exposures and outcomes. It is anticipated that the data will not allow for a meta-analysis and as such narrative synthesis will be used for each review question, and using the conceptual model referred to above to as a way to synthesise and illustrate the associations, mediators and moderators within the identified body of literature. The Synthesis Without Meta-analysis (SWiM) guidelines will be used to guide reporting of results [42]. To describe the associations between exposure and outcomes, studies will be grouped by exposure measure for synthesis. The quality assessment of individual studies will be used to determine the strength of the evidence and greater weight will be given to conclusions drawn from the most methodological sound and reliable studies. Summary tables will be produced for each grouping to describe the exposures, outcomes and effect sizes. Modifiers and mediators of the relationship will be described narratively using structured headings as determined by the participatory element of the review, as illustrated in the initial conceptual model (Fig. 1a).

This narrative synthesis will be used to generate findings and will inform a final diagram of the relationship between child development at primary school starting age and health outcomes in early adolescence.

### Additional analyses

Analysis by geographical context to capture any differences in the relationship by country will be considered during the data synthesis and will be identified in the narrative synthesis.

### Confidence in cumulative evidence

In addition to assessing the quality of each individual paper the overall strength of the review findings will be assessed drawing on criteria used by Hoogendoom [43] and Baxter [37, 44] together with principles of GRADE specific to observational studies [45] .The review findings by typology of papers, grouped by exposure, will be assessed for relative strength of evidence. The assessment will be based on volume, quality and consistency in effect sizes in studies. This will allow each review finding to be graded as either stronger, weaker, inconsistent or limited evidence. Assessment on the strength of evidence in relation to mediators and moderators of the relationship may be more difficult to grade using standard tools. Whereby any findings are based on theory papers or author opinion on proposed mechanisms this will be reflected in the grading of the evidence. Strength of evidence will also be illustrated in the final diagram. Agreement on grading of review findings will be agreed by the whole review team.

## Discussion

This review will address an important knowledge gap by increasing our understanding of the associations between measures of development and health in childhood, and the factors which affect these associations. By using participatory methods alongside systematic evidence synthesis the review will elucidate how children’s development at the age of starting school is related to subsequent adolescent health outcomes in contexts of socioeconomic inequality. This will inform ways to intervene to improve health and reduce health inequality in adolescents. The findings will generate knowledge of cross-sector relevance for health and education and promote inter-sectoral coherence in addressing health inequalities [46, 47] throughout childhood.

Any amendments made to this protocol when conducting the review will be outlined in PROSPERO and reported in the final manuscript. Results will be disseminated through conference presentations and publication in a peer-reviewed journal.

## Strengths and limitations

This review will provide, for the first time, a systematic overview of the association between child development at primary school entry, and adolescent health and factors that shape this relationship. It will incorporate stakeholder views to add depth and insight to guide the review process. The involvement of a sample of stakeholders raises the potential for biases to be introduced by selection of stakeholders with particular views, opinions or experiences. The risk of bias will be minimised by the use of transparent and replicable systematic review methods. The review may also be limited by primary studies with limited data on the mechanisms between exposure and outcome. Additionally, risk of bias in observational primary studies may bias the overall review results. This will be addressed at the quality assessment stage by recording risk of bias and using the assessment scores to decide the weight to assign to the conclusions drawn from each review. At review level, the heterogeneity of the study designs, exposure and outcome measures will need careful consideration in the data synthesis with care taken to group studies to ensure reliable and valid conclusions are drawn.

## Supplementary Information

**Additional file 1:.** PRISMA-P 2015 Checklist : Impact of child development at primary school entry on adolescent health – protocol for a participatory systematic review.

**Additional file 2:.** Search Terms–sample search strategy (Using obesity and mental health as example outcomes) for Impact of child development at primary school entry on adolescent health–protocol for a participatory systematic review.

**Additional file 3:.** Data Extraction Form

## Data Availability

Not applicable

## Abbreviations

DAG: Directed acyclic graph
GRADE: Grading of recommendations, assessment, development and evaluations
LQAT: Liverpool quality assessment tools
MCS: Millenium cohort study
OECD: Organisation for economic co-operation and development
PRISMA-P: Preferred reporting items for systematic review and meta-analysis protocols
PROSPERO: International prospective register of systematic reviews
SWiM: Synthesis without meta-analysis
