# Supplementary material for: Impact of child development at primary school entry on adolescent health—protocol for a participatory systematic review
Source: Syst Rev. 2021 May 7;10:142. doi: 10.1186/s13643-021-01694-6 (PMC8105931; doi:10.1186/s13643-021-01694-6)
Supplement: Supplementary file 2 — Additional file 2:. Search Terms–sample search strategy (Using obesity and mental health as example outcomes) for Impact of child development at primary school entry on adolescent health–protocol for a participatory systematic review. [file 13643_2021_1694_MOESM2_ESM.docx]

**Additional File 2: Search Terms – sample search strategy (Using obesity and mental health as example outcomes) for Impact of child development at primary school entry on adolescent health – protocol for a participatory systematic review.**

| Population | Exposure | Outcome |
| --- | --- | --- |
| Child* | School adj3 read* | Weight |
| Infant* | Early years and ed* | Obes* |
| Adolesc* | Early years and child dev* | Mental Health |
| Teen* | Pre-school | Socioemotional beahio* |
|  | Cognitive adj3 dev* | Social competence |
|  | Math |  |
|  | Writing |  |
|  | Test scores |  |
|  | Achiev* |  |
|  | Socioemo* |  |
|  | Behavio* |  |
|  | Physical dev* |  |

Database: Ovid MEDLINE(R) and Epub Ahead of Print, In-Process & Other Non-Indexed Citations and Daily <1946 to September 15, 2020>

Search Strategy:

--------------------------------------------------------------------------------

1 Child/ (1693243)

2 Adolescent/ (2034954)

3 Infant/ (793637)

4 Young Adult/ (857712)

5 teen*.mp. [mp=title, abstract, original title, name of substance word, subject heading word, floating sub-heading word, keyword heading word, organism supplementary concept word, protocol supplementary concept word, rare disease supplementary concept word, unique identifier, synonyms] (31116)

6 1 or 2 or 3 or 4 or 5 (3538881)

7 school read*.mp. [mp=title, abstract, original title, name of substance word, subject heading word, floating sub-heading word, keyword heading word, organism supplementary concept word, protocol supplementary concept word, rare disease supplementary concept word, unique identifier, synonyms] (741)

8 (early years and child dev*).mp. [mp=title, abstract, original title, name of substance word, subject heading word, floating sub-heading word, keyword heading word, organism supplementary concept word, protocol supplementary concept word, rare disease supplementary concept word, unique identifier, synonyms] (227)

9 (early years and ed*).mp. [mp=title, abstract, original title, name of substance word, subject heading word, floating sub-heading word, keyword heading word, organism supplementary concept word, protocol supplementary concept word, rare disease supplementary concept word, unique identifier, synonyms] (851)

10 pre-school.mp. [mp=title, abstract, original title, name of substance word, subject heading word, floating sub-heading word, keyword heading word, organism supplementary concept word, protocol supplementary concept word, rare disease supplementary concept word, unique identifier, synonyms] (4711)

11 (cognitve adj3 dev*).mp. [mp=title, abstract, original title, name of substance word, subject heading word, floating sub-heading word, keyword heading word, organism supplementary concept word, protocol supplementary concept word, rare disease supplementary concept word, unique identifier, synonyms] (0)

12 math*.mp. [mp=title, abstract, original title, name of substance word, subject heading word, floating sub-heading word, keyword heading word, organism supplementary concept word, protocol supplementary concept word, rare disease supplementary concept word, unique identifier, synonyms] (185812)

13 writing.mp. [mp=title, abstract, original title, name of substance word, subject heading word, floating sub-heading word, keyword heading word, organism supplementary concept word, protocol supplementary concept word, rare disease supplementary concept word, unique identifier, synonyms] (37834)

14 test score.mp. [mp=title, abstract, original title, name of substance word, subject heading word, floating sub-heading word, keyword heading word, organism supplementary concept word, protocol supplementary concept word, rare disease supplementary concept word, unique identifier, synonyms] (3201)

15 achiev*.mp. [mp=title, abstract, original title, name of substance word, subject heading word, floating sub-heading word, keyword heading word, organism supplementary concept word, protocol supplementary concept word, rare disease supplementary concept word, unique identifier, synonyms] (1060284)

16 abilit*.mp. [mp=title, abstract, original title, name of substance word, subject heading word, floating sub-heading word, keyword heading word, organism supplementary concept word, protocol supplementary concept word, rare disease supplementary concept word, unique identifier, synonyms] (939244)

17 socioemo*.mp. [mp=title, abstract, original title, name of substance word, subject heading word, floating sub-heading word, keyword heading word, organism supplementary concept word, protocol supplementary concept word, rare disease supplementary concept word, unique identifier, synonyms] (1781)

18 behaviour*.mp. [mp=title, abstract, original title, name of substance word, subject heading word, floating sub-heading word, keyword heading word, organism supplementary concept word, protocol supplementary concept word, rare disease supplementary concept word, unique identifier, synonyms] (289949)

19 physical dev*.mp. [mp=title, abstract, original title, name of substance word, subject heading word, floating sub-heading word, keyword heading word, organism supplementary concept word, protocol supplementary concept word, rare disease supplementary concept word, unique identifier, synonyms] (3168)

20 weight.mp. [mp=title, abstract, original title, name of substance word, subject heading word, floating sub-heading word, keyword heading word, organism supplementary concept word, protocol supplementary concept word, rare disease supplementary concept word, unique identifier, synonyms] (1093674)

21 obesity.mp. [mp=title, abstract, original title, name of substance word, subject heading word, floating sub-heading word, keyword heading word, organism supplementary concept word, protocol supplementary concept word, rare disease supplementary concept word, unique identifier, synonyms] (329062)

22 mental health.mp. [mp=title, abstract, original title, name of substance word, subject heading word, floating sub-heading word, keyword heading word, organism supplementary concept word, protocol supplementary concept word, rare disease supplementary concept word, unique identifier, synonyms] (196134)

23 socioemotional behaviour.mp. [mp=title, abstract, original title, name of substance word, subject heading word, floating sub-heading word, keyword heading word, organism supplementary concept word, protocol supplementary concept word, rare disease supplementary concept word, unique identifier, synonyms] (4)

24 social comp*.mp. [mp=title, abstract, original title, name of substance word, subject heading word, floating sub-heading word, keyword heading word, organism supplementary concept word, protocol supplementary concept word, rare disease supplementary concept word, unique identifier, synonyms] (5981)

25 20 or 21 or 22 or 23 or 24 (1501079)

26 7 or 8 or 9 (1724)

27 10 or 11 or 12 or 13 or 14 or 15 or 16 or 17 or 18 or 19 (2412026)

28 26 and 27 (616)

29 25 and 28 (92)
