# Supplementary material for: Impact of child development at primary school entry on adolescent health—protocol for a participatory systematic review
Source: Syst Rev. 2021 May 7;10:142. doi: 10.1186/s13643-021-01694-6 (PMC8105931; doi:10.1186/s13643-021-01694-6)
Supplement: Supplementary file 3 — Additional file 3:. Data Extraction Form [file 13643_2021_1694_MOESM3_ESM.docx]

**Additional File 3 – Data Extraction Form**

| **Authors:**  **Country:**  **Study objectives:**    **Study design :**   \| **Descriptive** \|  \| \| --- \| --- \| \| **RCT** \|  \| \| **Cohort** \|  \| \| **Sys. review** \|  \| \| **Longitudinal Analysis** \|  \| \| **Qualitative** \|  \| \| **Other:** \| \|   **Study population:**  **Contextual factors:**  **Data collection methods/sources:** | **Exposure and measurement instrument:**    **Outcomes and measurement instrument:**    **Health Inequalities/SES/Deprivation discussed?:**    **Mechanisms between exposure and outcome:**  **Moderators of the relationship between exposure and outcome:**  **Summary of Findings:** | **Main author conclusions:**    **Strengths:**    **Weaknesses:** |
| --- | --- | --- | --- | --- | --- | --- | --- | --- | --- | --- | --- | --- | --- | --- | --- | --- |
